# Population Serum 25-Hydroxyvitamin D Status in Kazakhstan: A Systematic Review and Meta-Analysis

**DOI:** 10.3390/diagnostics16121851

**Published:** 2026-06-15

**Authors:** Indira Karibayeva, Galiya Bilibayeva, Dinara Ospanova, Roza Alekesheva, Kaliya Kyzaikyzy, Zhanar Ibraimzhanova, Ainur Seitmanova, Zhanbota Sagyndyk, Gulden Bolatbekova, Aziza Bekenova

**Affiliations:** 1 Department of Research Management, JSC Research Institute of Cardiology and Internal Diseases, Almaty 050012, Kazakhstan; ik01379@georgiasouthern.edu; 2 Jiann-Ping Hsu College of Public Health, Georgia Southern University, Statesboro, GA 30460, USA; 3 Department of Nursing, Al-Farabi Kazakh National University, Almaty 050012, Kazakhstan; alekesheva@kaznu.kz (R.A.); kyzaikyzy@gmail.com (K.K.); ibraimzhanova.zh@kaznu.kz (Z.I.); seitmanova@kaznu.kz (A.S.); sagyndyk.zh@kaznu.kz (Z.S.); 4 Department of Fundamental Medicine, Al-Farabi Kazakh National University, Almaty 050012, Kazakhstan; guldenaibolatbekova@gmail.com; 5 Department of Normal Physiology with a Biophysics Course, Asfendiyarov Kazakh National Medical University, Almaty 050012, Kazakhstan; a.bekenova@kaznmu.kz

**Keywords:** vitamin D, 25-hydroxyvitamin D, Kazakhstan, serum vitamin D, systematic review, meta-analysis, public health, nutritional epidemiology

## Abstract

**Background/Objectives**: The aim of this study was to systematically synthesize and quantitatively estimate the mean serum 25-hydroxyvitamin D concentrations across populations in Kazakhstan and to examine variations according to age group, health status, and geographic region. In addition, we specifically evaluated healthy subgroups to establish reference estimates that may be useful for future epidemiological surveillance and public health planning. **Methods**: A systematic review and meta-analysis was conducted in accordance with the PRISMA 2020 guidelines. PubMed, Scopus, ScienceDirect, and Google Scholar were searched through March 2026 without date restrictions. Studies reporting the mean serum vitamin D concentrations among Kazakhstani populations were included. Random-effects meta-analysis was performed in R. Subgroup analyses were conducted by age group, health status, and geographic region. Meta-regression, influence diagnostics, publication bias assessment, JBI risk-of-bias evaluation, and GRADE certainty-of-evidence assessment were performed. **Results**: Sixteen studies comprising 28 groups and 5771 participants were included. The pooled mean serum 25(OH)D concentration in the overall cohort was 22.3 ng/mL (95% CI: 19.3–25.3), while the healthy cohort demonstrated a slightly higher pooled mean of 24.4 ng/mL (95% CI: 20.3–28.4). Adolescents had the lowest vitamin D levels among all age groups. Significant regional variability was observed, and meta-regression identified male participant proportion as a significant moderator (*p* = 0.03). Heterogeneity was extremely high across analyses (I^2^ ≈ 99.9%). **Conclusions**: Mean serum 25(OH)D concentrations were generally within the insufficient range across the included study groups in Kazakhstan, including healthy subgroups. However, because the certainty of evidence was very low and between-study heterogeneity was extreme, the findings should be interpreted cautiously. These results support the need for standardized national surveillance and locally evaluated public health policy considerations, including targeted supplementation for high-risk groups, screening strategies where clinically indicated, and assessment of food fortification options.

## 1. Introduction

Vitamin D is a fat-soluble secosteroid hormone that plays a central role in calcium and phosphorus homeostasis, skeletal development, immune regulation, and multiple metabolic processes [1]. Beyond its established role in bone health, vitamin D has been increasingly implicated in the pathophysiology of various chronic conditions [2,3]. Serum 25-hydroxyvitamin D [25(OH)D] is considered the most reliable biomarker of vitamin D status due to its relatively long half-life and reflection of both endogenous synthesis and dietary intake [4]. Despite its biological importance, vitamin D deficiency and insufficiency remain major global public health concerns. Globally, an estimated 15.7% (95% credible interval: 13.7–17.8) of the population had serum 25(OH)D concentrations below 30 nmol/L between 2000 and 2022, highlighting the substantial worldwide burden of severe vitamin D deficiency [5]. Regional variation is driven by latitude, climate, dietary habits, lifestyle, and socioeconomic factors [6,7]. Countries located at higher latitudes or characterized by prolonged winter seasons are particularly vulnerable to widespread vitamin D insufficiency due to reduced ultraviolet B exposure.

The health consequences of vitamin D insufficiency differ across the life course but remain clinically significant in both children and adults. In children and adolescents, insufficient vitamin D levels are associated with impaired bone mineralization, rickets, reduced peak bone mass acquisition, impaired immune function, and potentially adverse neurodevelopmental outcomes [8,9]. Adolescence represents a particularly vulnerable period due to rapid skeletal growth and increased physiological demand [10]. In adults, vitamin D insufficiency has been associated with osteoporosis, muscle weakness, cardiometabolic disorders, chronic inflammation, immune dysregulation, and increased susceptibility to infectious and chronic diseases [2,3]. Although the causal relationships between vitamin D and some non-skeletal outcomes remain debated, persistent low serum 25(OH)D levels are widely recognized as an important marker of population health vulnerability. Consequently, understanding the distribution of serum vitamin D concentrations across age groups and health conditions is essential for developing evidence-based prevention and supplementation strategies.

Kazakhstan is a geographically large country located in Central Asia, with approximate geographic coordinates of 48° N and 68° E. The country has substantial topographic and climatic heterogeneity, with marked variation from low-lying areas near the Caspian Basin to high mountain systems in the south and east [11]. Kazakhstan has a strongly continental climate, characterized by cold winters, hot summers, and arid to semi-arid conditions [11,12]. These environmental features are directly relevant to vitamin D status because seasonal ultraviolet B availability, outdoor exposure patterns, temperature, precipitation, and cloud cover may influence cutaneous vitamin D synthesis. Population distribution may also be relevant: at the beginning of 2025, Kazakhstan had 20,283,399 residents, including 12,773,115 urban residents and 7,510,284 rural residents, corresponding to an approximately 63.0% urban and 37.0% rural population distribution [13]. Urban–rural residence may influence sunlight exposure, outdoor activity, diet, socioeconomic status, and access to supplementation. Skin phototype is another relevant determinant of cutaneous vitamin D synthesis. Kazakhstan is a multinational and multiethnic country, and ethnic Kazakhs themselves are phenotypically heterogeneous, reflecting the mixed East Eurasian and West Eurasian ancestry of the Kazakh population. Therefore, the population cannot be assigned a single Fitzpatrick skin type. Available regional evidence suggests that Fitzpatrick types II–III may be common in Kazakhstan-based cohorts, while broader Asian populations are often described within types III–IV [14].

In Kazakhstan, emerging evidence indicates that vitamin D insufficiency is highly prevalent across both pediatric and adult populations. Recent meta-analyses demonstrated that the pooled prevalence of vitamin D deficiency among children in Kazakhstan was 56%, with even higher rates among infants at 65% [15]. Similarly, vitamin D deficiency among adults was highly prevalent, affecting approximately 60% of adults with chronic conditions and 55% of apparently healthy adults [16]. However, prevalence estimates alone provide limited insight into the underlying distribution and severity of vitamin D insufficiency within the population. Mean serum 25(OH)D concentrations represent an important complementary indicator because they allow for a quantitative assessment of population vitamin D status, facilitate comparisons across demographic and geographic groups, and provide a measurable baseline for future public health monitoring.

Therefore, the aim of this study was to systematically synthesize and quantitatively estimate the mean serum 25(OH)D concentrations across populations in Kazakhstan and to examine variations according to age group, health status, and geographic region. In addition to assessing the overall population, we specifically evaluated healthy subgroups to establish reference estimates that may be useful for future epidemiological surveillance and public health planning.

## 2. Methods

The PROSPERO International Prospective Register of Systematic Reviews database was searched to identify the registration of similar reviews, and none were found. Therefore, the current study protocol was registered with PROSPERO (ID: CRD42024610447) (https://www.crd.york.ac.uk/PROSPERO/view/CRD42024610447, accessed on 10 May 2026).

### 2.1. Search Strategy, Eligibility Screening, and Data Extraction

We conducted a systematic search of four major electronic databases: PubMed, Scopus, ScienceDirect, and Google Scholar. The search strategy was developed in advance, and in accordance with the Preferred Reporting Items for Systematic Reviews and Meta-Analyses 2020 (PRISMA) guidelines [17], two independent reviewers (I.K. and G.Bi.) performed the literature search after reaching agreement on the final search strategy across all databases. No date restrictions were applied, and the search was completed in March 2026. The PRISMA checklist is provided in Supplementary Table S1.

The search strategy included the keywords “vitamin D” and “Kazakhstan”. Database-specific filters were applied where appropriate: in PubMed, filters for English language and human subjects were used; in Scopus, results were limited to articles published in English involving human subjects; and in ScienceDirect, searches were restricted to research articles. Searches in Google Scholar were conducted using title-only queries to improve specificity.

Methodologically, literature screening and synthesis of the search results also adhered to the PRISMA guidelines [17]. The inclusion criteria were as follows: (a) Studies reporting mean and standard deviation (SD) values of serum 25-hydroxyvitamin D or vitamin D levels among the Kazakhstani population; (b) Cohort studies, cross-sectional studies, and case–control studies; (c) Studies involving adults, adolescents, and children residing in Kazakhstan. The exclusion criteria were as follows: (a) Studies that duplicated previously presented results; (b) Studies reporting vitamin D deficiency or insufficiency prevalence; (c) Case reports, reviews, editorials, and non-original research articles; (d) Studies published in languages other than English.

Following the PRISMA guidelines, two authors (I.K. and G.Bi.) independently extracted the required data from the included full-text articles using a standardized data extraction form. The extracted variables included: (1) first author’s name, (2) publication year, (3) study region, (4) study design, (5) sample size, (6) number of male participants, (7) age, (8) mean ± standard deviation (SD) of serum 25-hydroxyvitamin D levels (ng/mL; all values were converted to ng/mL where necessary), (9) population group (healthy or specific clinical condition), and (10) population category (adults, adolescents, or children). Any discrepancies were resolved through discussion and consensus with a third author. No missing data were imputed for further data analysis.

The main outcome of interest was the mean serum 25(OH)D concentrations. In the present review, vitamin D status terminology was standardized as follows: serum 25(OH)D concentrations < 20 ng/mL were considered vitamin D deficiency, concentrations of 20.0–29.9 ng/mL were considered vitamin D insufficiency, and concentrations ≥ 30 ng/mL were considered vitamin D sufficiency.

### 2.2. Risk of Bias and Certainty of Evidence Assessment

Risk of bias was assessed using the Joanna Briggs Institute (JBI) Critical Appraisal Checklist for Analytical Cross-Sectional Studies. This instrument evaluates key methodological domains, including clearly defined inclusion criteria, description of study participants and setting, valid and reliable exposure measurement, objective and standardized outcome measurement, identification and management of confounding factors, and appropriateness of statistical analysis (Q1 to Q8). Each item was rated as “yes”, “no”, “unclear”, or “not applicable”. For summary scoring, “yes” responses were assigned 1 point, while “no” and “unclear” responses were assigned 0 points; items marked “not applicable” were excluded from the denominator. Studies scoring ≥ 70% of applicable items were considered to have a low risk of bias, scores of 50–69% indicated moderate risk, and scores < 50% indicated high risk of bias. Two reviewers (I.K. and G.Bi.) independently performed the risk-of-bias assessment, and any discrepancies were resolved through discussion with a third reviewer (D.O.).

The overall certainty of the evidence was assessed using the Grading of Recommendations Assessment, Development and Evaluation (GRADE) approach. The certainty was evaluated across key domains, including risk of bias, inconsistency, indirectness, imprecision, and publication bias. Given the observational nature of the included studies, the evidence was initially rated as low certainty and subsequently downgraded or upgraded based on these domains. The overall certainty of evidence for each outcome was categorized as high, moderate, low, or very low, in accordance with the GRADE guidelines.

### 2.3. Statistical Analysis

Statistical analyses were performed using R (R Foundation for Statistical Computing, Vienna, Austria; version 4.5.1) within the RStudio environment (Posit Software, PBC, Boston, MA, USA; version 2025.9.0.387) [18,19]. The meta and metafor packages were used to calculate the pooled mean serum vitamin D levels with 95% confidence intervals (CIs). All available groups of children, adolescents, and adults were initially included in the analysis. A secondary analysis was conducted including only healthy subgroups. A random-effects model was applied throughout. Forest plots were generated to present pooled estimates using the “RevMan5” layout. Between-study heterogeneity was assessed using meta-regression, with the proportion of male participants included as a moderator. Influence diagnostics were performed to evaluate the robustness of the pooled estimates. Publication bias was assessed through visual inspection of funnel plots and statistically using Egger’s test. Subgroup analyses were conducted by population category (children, adolescents, and adults). Additional analyses were performed by geographic region within Kazakhstan.

## 3. Results

### 3.1. Description of the Included Studies

**Figure 1:**
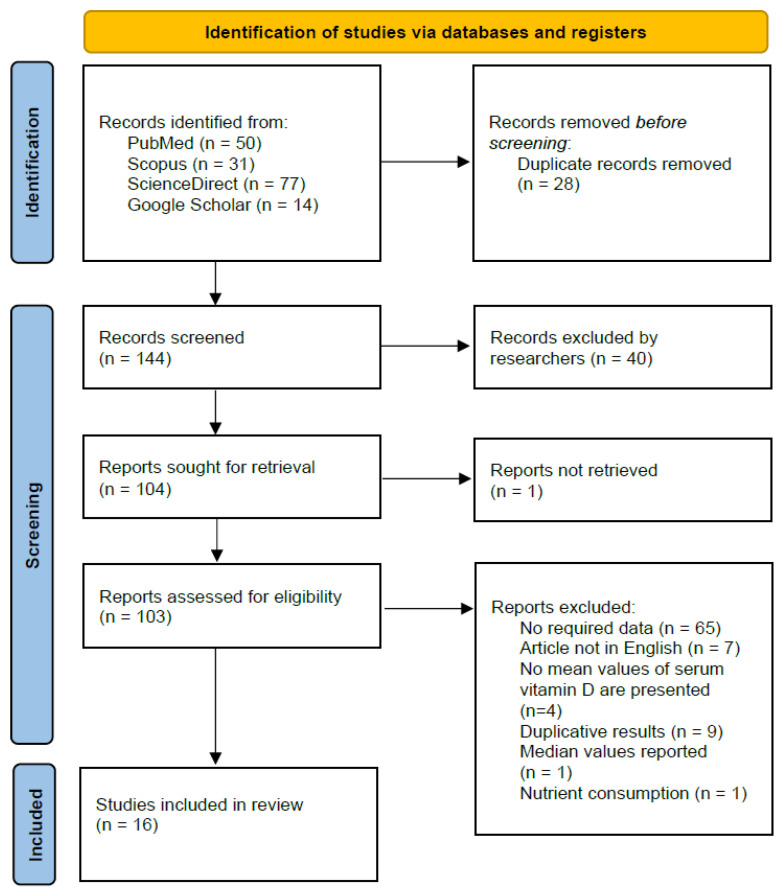
PRISMA flowchart of study selection [17].

The initial database search yielded a total of 172 results. After removing 28 duplicates and 40 ineligible studies, 104 full-text studies were selected for eligibility screening. Sixteen studies with twenty eight groups were included in the present systematic review and meta-analysis. Studies with overlapping or potentially similar populations were excluded to avoid the duplication of data [20,21,22,23,24,25,26,27,28]. Additionally, one study focusing on nutrient consumption [29] and one study reporting median vitamin D values [30] were excluded. A detailed flowchart of the study selection with the exclusion criteria process is depicted in Figure 1 [17].

The study characteristics, presented in Table 1, are divided into two groups of patients: children and adolescents and adults. The included studies were published between 2014 and 2025 and originated from various regions of Kazakhstan, including Almaty, Astana, Karaganda, Aktobe, Abay, and the Almaty and Akmola oblasts. Five studies assessed vitamin D levels among 1248 children and adolescents across ten groups. Eleven studies assessed the vitamin D levels of 4523 adults across eighteen groups.

### 3.2. Pooled Mean 25-Hydroxyvitamin D Levels in Kazakhstan

**Figure 2:**
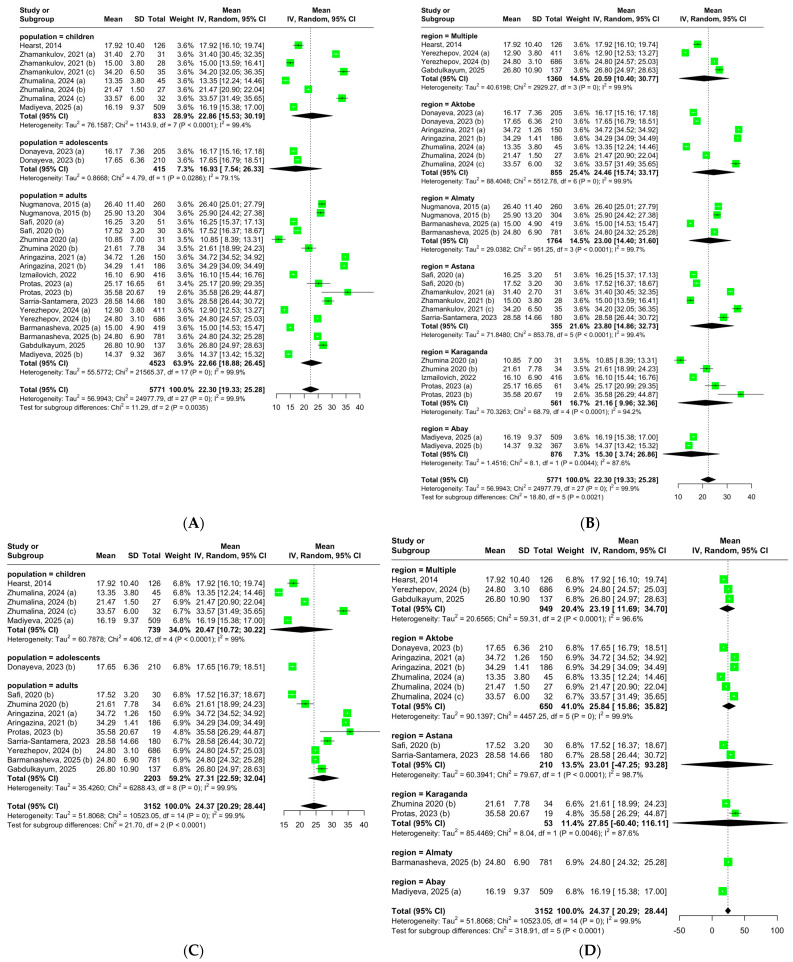
Pooled mean serum vitamin D level in Kazakhstan: (**A**) Overall cohort by population; (**B**) overall cohort by region; (**C**) healthy cohort by population; (**D**) healthy cohort by region. Studies included in the meta-analysis and description of groups: Hearst, 2014 [31]; Zhamankulov, 2021 [32]: With RRI and atopic phenotype (a); D-deficiency phenotype (b); RRI with no D def or atopic phenotype (c); Donayeva, 2023 [33]: Primary dysmenorrhea (a); Healthy (b); Zhumalina, 2024 [34]: 0–28 days (a); 1–6 months (b); 7–12 months (c); Madiyeva, 2025 (a) [35]; Nugmanova, 2015 [36]: HIV—viral load detectable (a); HIV—viral load undetectable (b); Safi, 2020 [37]: PCOS (a); Healthy (b); Zhumina, 2020 [38]: Leukemia (a); Healthy (b); Aringazina, 2021 [39]: Healthy (placebo group) (a); Healthy (treatment group) (b); Izmailovich, 2022 [40]; Protas, 2023 [41]: COVID-19 (a); Healthy (b); Sarria-Santamera, 2023 [42]; Yerezhepov, 2024 [43]: Tuberculosis (a); Healthy (b); Barmanasheva, 2025 [44]: Uterine fibroid (a); Healthy (b); Gabdulkayum, 2025 [45]; Madiyeva, 2025 (b) [46].

Figure 2 presents the pooled mean serum 25-hydroxyvitamin D [25(OH)D] concentrations across the included studies. In the overall cohort (Figure 2A,B), the pooled mean vitamin D level was 22.3 ng/mL (95% CI: 19.3–25.3), indicating generally insufficient vitamin D status at the population level. However, heterogeneity across studies was extremely high (I^2^ ≈ 99.9%, *p* < 0.001), suggesting substantial variability beyond chance. When stratified by population group (Figure 2A), the mean vitamin D levels were lowest among adolescents (~16 ng/mL), followed by adults (~22 ng/mL), and slightly higher in children (~22 ng/mL), although all subgroups remained within insufficient ranges. Subgroup differences were statistically significant, indicating that age-related physiological and behavioral factors may contribute to variability in vitamin D status. Regional analysis (Figure 2B) further demonstrated notable variability, with higher pooled estimates observed in the central, west and southern regions compared to the east regions (e.g., Abay). These differences likely reflect geographic variation in latitude, sun exposure, and environmental factors.

In the healthy population (Figure 2C,D), the pooled mean vitamin D level was slightly higher at 24.4 ng/mL (95% CI: 20.3–28.4), although still within the insufficient range. Heterogeneity remained very high (I^2^ ≈ 99.9%, *p* < 0.001), indicating that restricting the analysis to healthy individuals did not meaningfully reduce between-study variability. Compared to the overall cohort, the healthy population demonstrated higher mean vitamin D levels, particularly among adults, suggesting that comorbid conditions (e.g., HIV infection, tuberculosis, COVID-19, PCOS, and malignancies) contribute to lower vitamin D levels in the general pooled estimates. Nevertheless, adolescents had the lowest mean serum 25(OH)D concentrations and were the subgroup most consistently falling below sufficiency thresholds, including in the healthy-population analysis. Overall, the observed heterogeneity is likely driven by a combination of factors, including population characteristics (age, health status), geographic variability (latitude and region), study design differences, seasonal variation, and measurement methods. These findings highlight the substantial variability in vitamin D status across Kazakhstan and underscore the importance of stratified analyses in interpreting pooled estimates.

Meta-regression analysis based on the proportion of male participants indicated significant variation in effect sizes according to gender distribution. The analysis showed that studies with a higher proportion of male participants reported larger effect sizes (*p* = 0.03). The fitted meta-regression equation was:

*Mean serum 25(OH)D = 17.8 + 12.7 × proportion of male participants.*

**Figure 3:**
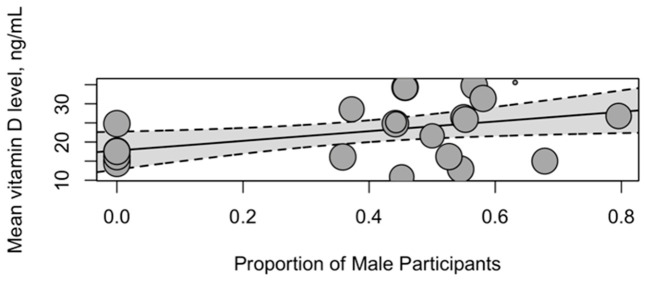
Meta-regression analysis by the proportion of male participants.

The results of the meta-regression are presented in Figure 3.

**Figure 4:**
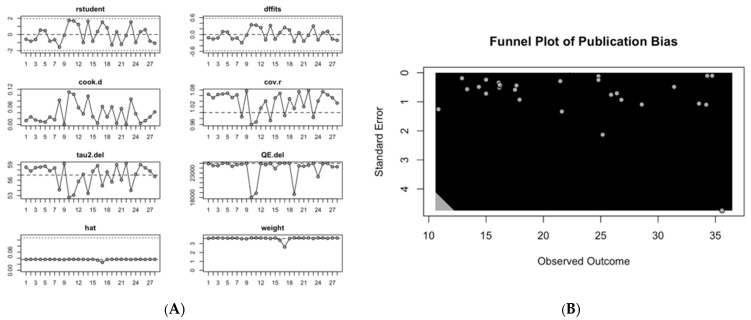
Influence diagnostics (**A**) and funnel plot for publication bias (**B**).

Figure 4 presents the influence diagnostics (Panel A) and funnel plot for publication bias (Panel B). The influence analysis did not identify any single study exerting a disproportionate effect on the pooled estimates. Standardized residuals, Cook’s distances, and leverage values were generally within acceptable ranges, indicating that the overall results were robust and not driven by outliers or highly influential studies. Visual inspection of the funnel plot (Figure 4B) showed a relatively symmetrical distribution of effect sizes around the pooled estimate, suggesting no substantial small-study effects. This observation was further supported by Egger’s test, which was not statistically significant (*p* > 0.05), indicating no evidence of publication bias.

Overall, these findings support the stability and reliability of the pooled estimates despite the high between-study heterogeneity observed in the meta-analysis.

### 3.3. Risk of Bias Evaluation and Certainty of Evidence Assessment

The risk of bias assessment using JBI Critical Appraisal Checklist indicated that all included studies were of low methodological risk (Table 2). The majority of studies achieved high scores, with 8/8 criteria fulfilled in seven studies, 7/8 in six studies, and 6/8 in three studies, reflecting generally strong methodological quality across the evidence base. Most studies clearly defined their study populations and settings, employed objective and standardized measurement of serum 25-hydroxyvitamin D, and used appropriate statistical analyses. Studies involving clinical populations, including individuals with HIV, tuberculosis, COVID-19, and PCOS, demonstrated particularly robust design features, often incorporating multivariable analytical approaches and well-defined comparison groups. Minor methodological limitations were identified in several studies. These primarily related to incomplete adjustment for potential confounding factors, limited reporting of confounder control strategies, or small sample sizes in pilot or subgroup analyses. In pediatric and community-based studies, confounding related to environmental and behavioral factors was not consistently addressed. Similarly, some retrospective and subgroup analyses lacked full transparency regarding participant selection or exposure classification. Despite these limitations, the overall body of evidence was considered methodologically sound. No studies were judged to have a high risk of bias.

The GRADE assessment indicated that the overall certainty of evidence for pooled serum 25-hydroxyvitamin D levels in both the overall and healthy cohorts was very low (Table 3). Although the risk of bias, indirectness, imprecision, and publication bias were not considered serious, the evidence was downgraded by two levels because of very serious inconsistency, reflected by substantial between-study heterogeneity. These findings suggest that although the pooled estimates are informative, they should be interpreted with caution due to the high variability across studies.

## 4. Discussion

This systematic review and meta-analysis provide a comprehensive synthesis of the mean serum 25(OH)D concentrations reported in studies conducted in Kazakhstan. The pooled mean level across all included study groups was 22.3 ng/mL, and the pooled estimate among healthy subgroups was 24.4 ng/mL, both of which fall within commonly used insufficiency ranges. However, these estimates should be interpreted cautiously. The overall analysis included both healthy and clinical or selected populations, including participants with HIV, tuberculosis, COVID-19, PCOS, leukemia, uterine fibroids, somatic diseases, and elite athletes. Therefore, the overall pooled estimate should not be interpreted as a nationally representative estimate of serum 25(OH)D status in the entire population of Kazakhstan. Although the secondary healthy-subgroup analysis provides a more relevant reference estimate, it also cannot be considered nationally representative. Rather, the findings indicate that suboptimal serum 25(OH)D concentrations are consistently observed across available study populations and highlight the need for standardized, nationally representative vitamin D surveillance.

A key strength of this study is the identification of systematic variation across age groups, with adolescents demonstrating the lowest vitamin D levels. This finding is consistent with global epidemiological patterns and highlights adolescence as a critical window of vulnerability [47,48]. Behavioral factors, including reduced outdoor activity, increased indoor sedentary behavior, and suboptimal dietary intake, likely contribute to this pattern [49]. In addition, increased physiological demands during growth may further exacerbate deficiency [50]. The persistence of insufficient levels even among healthy adolescents underscores the need for targeted preventive strategies in this population.

Although adolescents demonstrated the lowest pooled serum 25(OH)D concentrations in this review, the adult findings are also clinically and public-health relevant. Most adult subgroups had mean serum 25(OH)D concentrations below commonly used sufficiency thresholds, suggesting that suboptimal vitamin D status is not limited to pediatric or adolescent populations. In adults, low serum 25(OH)D has been associated in observational studies with several outcomes relevant to population health, including poorer skeletal health, muscle weakness and falls, cardiometabolic disorders, immune dysregulation, chronic inflammation, respiratory infections, and higher all-cause and cause-specific mortality risks [51]. These associations are particularly important in Kazakhstan, where noncommunicable diseases account for a substantial share of adult morbidity and mortality, and where cardiovascular diseases, cerebrovascular disease, chronic respiratory diseases, and cancer remain major contributors to mortality [52,53].

The findings also highlight the role of geographic and environmental determinants. Kazakhstan’s high latitude and continental climate, characterized by long winters and limited ultraviolet B exposure, are likely major contributors to low endogenous vitamin D synthesis [54]. Regional differences observed in this study support this interpretation, although some unexpected patterns suggest that unmeasured population-level factors, such as occupational exposure, urbanization, or socioeconomic differences, may also influence vitamin D status. These findings emphasize that geographic variation cannot be interpreted solely through latitude but must be considered within a broader environmental and social context [55].

Dietary patterns may further contribute to the observed insufficiency. Traditional dietary practices in Kazakhstan include a substantial consumption of meat and animal products, which may provide some vitamin D and 25-hydroxyvitamin D metabolites [56]. However, meat alone is unlikely to fully compensate for limited ultraviolet B exposure, low oily-fish intake, and the absence of widespread vitamin D fortification [57]. In contrast to many high-income countries where vitamin D fortification is widespread, Kazakhstan currently lacks a comprehensive fortification strategy [58]. This structural gap likely contributes not only to the persistence of suboptimal vitamin D levels, but also to broader micronutrient deficiencies at the population level and suggests a clear opportunity for policy intervention.

Our results provide important additional insight by identifying gender as a significant factor, with higher proportions of male participants associated with higher vitamin D levels. This finding is biologically and behaviorally plausible, as men may have greater sun exposure and lower adiposity-related sequestration of vitamin D [59,60]. Additionally, studies suggest that sex hormones influence the production, metabolism, and bioavailability of vitamin D, which may further contribute to sex-related differences in serum 25(OH)D concentrations [61,62]. The identification of gender as a modifier underscores the importance of considering demographic composition when interpreting pooled estimates and designing interventions. More broadly, the persistence of extreme heterogeneity across analyses suggests that vitamin D status in Kazakhstan is shaped by a complex interplay of individual, behavioral, environmental, and systemic factors.

These findings can be interpreted within a social-ecological framework [63], where health determinants operate at multiple levels. At the individual level, age and sex differences are evident. At the behavioral level, lifestyle factors such as physical activity and diet influence vitamin D exposure. Socioeconomic factors such as household income operate at both the interpersonal and structural levels, influencing dietary quality, access to supplementation, healthcare utilization, and opportunities for sunlight exposure [64]. At the environmental level, latitude and climate limit ultraviolet radiation. At the system level, the absence of food fortification policies and limited public awareness contribute to persistent insufficiency. The high heterogeneity observed in this study reflects the interaction of these factors and highlights the need for multilevel interventions rather than single-factor solutions.

Comparison with international evidence suggests that the magnitude of vitamin D insufficiency in Kazakhstan is consistent with that observed in other regions with similar climatic and socio-environmental characteristics, including Central Asia and Eastern Europe [65,66]. However, the persistence of insufficient levels even among healthy individuals suggests that environmental exposure alone is insufficient to maintain adequate vitamin D status in this context. This contrasts with some higher-income settings, where fortification and supplementation programs have mitigated deficiency despite similar climatic conditions [67,68,69]. Evidence from previous systematic reviews indicates that food fortification with vitamin D is associated with improved dietary vitamin D intake and increased serum vitamin D concentrations [70,71].

The comparison between the overall cohort and the healthy subgroups suggests that suboptimal serum 25(OH)D concentrations are not limited to clinical populations. Although clinical populations exhibited slightly lower levels, the difference was modest, indicating that chronic disease may exacerbate deficiency but does not fully explain its prevalence. These findings may inform public health planning by identifying the need for improved surveillance and risk-stratified prevention strategies, but they do not provide sufficient evidence to determine whether universal, targeted, or combined intervention approaches would be most appropriate for Kazakhstan.

Seasonality is a particularly important determinant of serum 25(OH)D concentrations in countries with marked climatic variation. In Kazakhstan, where long winters and seasonal variation in ultraviolet B exposure may reduce cutaneous vitamin D synthesis, serum 25(OH)D concentrations are likely to fluctuate across the year. Previous mechanistic studies suggest that skeletal muscle may contribute to the maintenance of circulating 25(OH)D during winter by storing vitamin D metabolites and releasing them back into circulation, partly attenuating seasonal declines [72,73]. Nevertheless, winter serum 25(OH)D concentrations may remain substantially lower than summer concentrations in populations with limited ultraviolet B exposure [74]. In the present review, seasonality could not be quantitatively evaluated because the included studies did not consistently report month or season of blood sampling or provide season-specific 25(OH)D estimates. Therefore, seasonal variation should be considered an important unmeasured contributor to heterogeneity and a priority variable for future vitamin D surveillance studies in Kazakhstan.

International biomarker surveillance demonstrates that population vitamin D status is modifiable over time. Recent analyses of repeated U.S. National Health and Nutrition Examination Survey data have shown that serum 25(OH)D concentrations and the distribution of vitamin D status can change at the population level over extended periods, even in the context of concurrent changes in obesity prevalence [75]. These findings suggest that population-level monitoring, public awareness, supplement use, and food or nutrition policies may influence vitamin D status over time. However, such evidence should be interpreted as context rather than direct evidence for Kazakhstan. Kazakhstan currently lacks comparable long-term national biomarker surveillance of serum 25(OH)D, and the available studies included in this review were heterogeneous in design, population, region, season, and reporting quality. Therefore, our findings support the need for standardized national monitoring and carefully evaluated prevention strategies.

Recommendations for vitamin D intake and supplementation vary across countries and organizations, reflecting differences in latitude, diet, fortification policy, age structure, sun exposure, and interpretation of the evidence. In the United States, the National Academies’ dietary reference intakes recommend 600 IU/day for most adults aged 19–70 years and 800 IU/day for adults aged ≥71 years [76]. In the United Kingdom, national public health guidance recommends that adults and children over 4 years should consider taking 10 µg/day (400 IU/day) of vitamin D during autumn and winter, with year-round supplementation advised for individuals at higher risk of deficiency [77]. The Nordic Nutrition Recommendations recommend 10 µg/day for most adults and 20 µg/day for adults aged ≥75 years and also recommend 20 µg/day for individuals with little or no sun exposure [78]. The 2024 Endocrine Society guideline further suggests empiric vitamin D supplementation above the usual recommended intake for selected groups, including children and adolescents aged 1–18 years, adults older than 75 years, pregnant individuals, and adults with high-risk prediabetes, while advising against routine 25(OH)D testing in generally healthy adults without established indications [79]. These international recommendations provide useful context for Kazakhstan, but they should not be transferred directly without local adaptation. Kazakhstan differs in climate, diet, food fortification infrastructure, healthcare access, ethnicity and skin phototype distribution, and population-level risk profiles.

Several limitations of this review must be acknowledged. First, the included studies were predominantly cross-sectional, limiting causal inference and increasing susceptibility to residual confounding. Second, although the risk of bias was generally low, important confounders such as seasonal variation, dietary intake, and socioeconomic status were not consistently measured or adjusted for. Third, the extremely high heterogeneity represents a key limitation, reducing the precision of pooled estimates despite consistent overall trends. Fourth, variability in laboratory assays may have introduced measurement differences across studies. Another important limitation is that the original studies did not consistently report important confounders such as season of blood sampling, cloud cover, ultraviolet exposure, urban–rural residence, altitude, supplement use, Fitzpatrick skin phototype, age-stratified serum 25(OH)D estimates, BMI, obesity prevalence, pregnancy/lactation status, or BMI-stratified vitamin D values; therefore, these potentially important determinants could not be incorporated into additional quantitative analyses. Finally, the GRADE assessment indicated very low certainty of evidence, primarily due to very serious inconsistency, highlighting the need for more standardized and methodologically rigorous research.

## 5. Conclusions

This systematic review and meta-analysis indicate that mean serum 25(OH)D concentrations are generally within the insufficient range across available study groups in Kazakhstan, including both healthy subgroups and clinical or selected populations. Adolescents had the lowest mean serum 25(OH)D concentrations, and variability by gender composition and region suggests that vitamin D status is likely influenced by demographic, environmental, behavioral, and systemic factors. However, the findings should be interpreted cautiously because the certainty of evidence was very low, heterogeneity was extreme, and the included studies were not nationally representative.

From a public health perspective, these findings support the need for a standardized national surveillance of serum 25(OH)D concentrations in Kazakhstan. They also provide a rationale for considering locally adapted prevention strategies, including targeted supplementation for high-risk groups, clinically indicated screening, public awareness, and the evaluation of food fortification options. However, such strategies should be regarded as public health policy considerations requiring further local evidence, feasibility assessment, and monitoring rather than direct evidence-based recommendations derived from the present meta-analysis alone.

## Figures and Tables

**Table 1:** Summary of included studies.

Author, Year	Region	Study Design	Healthy/Disease Name	Cohort	Age (Mean ± SD)	Number of Male Patients
Children & Adolescents						
Hearst, 2014 [31]	Almaty, Astana, and Karaganda cities; Almaty and Akmola oblasts	Cross-sectional	Healthy	126	0–3 years	Not provided
Zhamankulov, 2021 [32]	Astana	Cross-sectional	With RRI and atopic phenotype (a)	31	6.4 ± 3.8	18
			D-deficiency phenotype (b)	28	5.8 ± 3.3	19
			RRI with no D def or atopic phenotype (c)	35	5.4 ± 3.2	16
Donayeva, 2023 [33]	Aktobe	Cross-sectional	Primary dysmenorrhea (a)	205	14.8 ±1.7	0
			Healthy (b)	210	15.2 ±1.6	0
Zhumalina, 2024 [34]	Aktobe	Cross-sectional	Healthy (a)	45	0–28 days	Not provided
			Healthy (b)	27	1–6 months	Not provided
			Healthy (c)	32	7–12 months	Not provided
Madiyeva, 2025 [35]	Abay	Cross-sectional	Healthy (a)	509	10.7 ± 3.4	268
Adults						
Nugmanova, 2015 [36]	Almaty	Cross-sectional	HIV—viral load detectable (a)	260	35.9 ± 8.7	143
			HIV—viral load undetectable (b)	304	38.2 ± 9.0	168
Safi, 2020 [37]	Astana	Cross-sectional	PCOS (a)	51	18–44	0
			Healthy (b)	30	18–44	0
Zhumina, 2020 [38]	Karaganda	Cross-sectional	Leukemia (a)	31	57.7 ± 13.8	14
			Healthy (b)	34	54.7 ± 15.1	17
Aringazina, 2021 [39]	Aktobe	Cross-sectional	Healthy (placebo group) (a)	150	56.3 ± 2.4	85
			Healthy (treatment group) (b)	186	56.3 ± 2.4	85
Izmailovich, 2022 [40]	Karaganda	Cross-sectional	Allergic rhinitis	416	39±8	149
Protas, 2023 [41]	Karaganda	Cross-sectional	COVID-19 (a)	61	43 ± 14.4	27
			Healthy (b)	19	41 ± 15.2	12
Sarria-Santamera, 2023 [42]	Astana	Cross-sectional	Healthy	180	41.9 ± 13.2	67
Yerezhepov, 2024 [43]	3 regions of Kazakhstan	Case–control	Tuberculosis (a)	411	35 ± 13.1	224
			Healthy (b)	686	40.5 ± 13.9	303
Barmanasheva, 2025 [44]	Almaty	Cross-sectional	Uterine fibroid (a)	419	42.5 ± 7.9	0
			Healthy (b)	781	39.8 ± 7.6	0
Gabdulkayum, 2025 [45]	2 regions of Kazakhstan	Cross-sectional	Healthy elite athletes	137	23.7 ± 4.3	109
Madiyeva, 2025 [46]	Abay	Cross-sectional	Somatic diseases (b)	367	69.8 ± 18.2	0

Abbreviations: COVID-19—Coronavirus Disease 2019; D-deficiency—Vitamin D deficiency; HIV—Human Immunodeficiency Virus; PCOS—Polycystic Ovary Syndrome; RRI—Recurrent Respiratory Infection; SD—Standard Deviation.

**Table 2:** JBI Risk of bias evaluation results.

Author, Year	Study Design/Population	Q1	Q2	Q3	Q4	Q5	Q6	Q7	Q8	Score	Risk of Bias	Main Reason for Judgement
Hearst, 2014 [31]	Cross-sectional; children in baby houses	Y	Y	Y	Y	U	N	Y	Y	6/8	Low	Clear child sample and biomarker outcome, but limited adjustment for confounding.
Nugmanova, 2015 [36]	Cross-sectional; adults with HIV	Y	Y	Y	Y	Y	Y	Y	Y	8/8	Low	Well-described clinical sample with multivariable analyses.
Safi, 2020 [37]	Comparative cross-sectional; PCOS and controls	Y	Y	Y	Y	Y	Y	Y	Y	8/8	Low	Clearly defined groups, laboratory measurement, and appropriate comparative analyses.
Zhumina, 2020 [38]	Observational case–control/cross-sectional; leukemia and controls	Y	Y	Y	Y	Y	U	Y	Y	7/8	Low	Groups and biomarker measurement were clear; residual confounding control was limited.
Zhamankulov, 2021 [32]	Retrospective cross-sectional; children with RRI and COVID-19	Y	Y	Y	Y	Y	U	Y	Y	7/8	Low	Clinical grouping was defined; strategies for confounding were only partly described.
Aringazina, 2021 [39]	Intervention/controlled follow-up; adults receiving vitamin D3 or placebo	Y	Y	Y	Y	Y	U	Y	Y	7/8	Low	Outcome and intervention groups were clear; allocation/confounding details were not fully transparent for this review outcome.
Izmailovich, 2022 [40]	Cross-sectional; seasonal allergic rhinitis	Y	Y	Y	Y	Y	U	Y	Y	7/8	Low	Large clinical sample and objective 25(OH)D assay; limited confounding adjustment.
Donayeva, 2023 [33]	Cross-sectional; primary dysmenorrhea subgroup	Y	Y	Y	Y	Y	U	Y	Y	7/8	Low	Outcome and clinical phenotype were defined; residual confounding may remain.
Protas, 2023 [41]	Pilot retrospective comparative study; COVID-19 positive/negative	Y	Y	Y	Y	U	N	Y	Y	6/8	Low	Objective laboratory outcome, but small pilot design and limited confounding control.
Sarria-Santamera, 2023 [42]	Case–control/cross-sectional; asymptomatic COVID-19 susceptibility	Y	Y	Y	Y	Y	U	Y	Y	7/8	Low	Study population and genotyping were described; exposure history/confounding remained partly uncertain.
Yerezhepov, 2024 [43]	Case–control; pulmonary tuberculosis and controls	Y	Y	Y	Y	Y	Y	Y	Y	8/8	Low	Well-described recruitment, exclusions, objective assay, and adjusted analyses.
Zhumalina, 2024 [34]	Cross-sectional; infants/young children	Y	Y	Y	Y	U	N	Y	Y	6/8	Low	Age strata and vitamin D outcome were clear; confounding control was limited.
Barmanasheva, 2025 [44]	Prospective facility-based cross-sectional; women of reproductive age	Y	Y	Y	Y	Y	Y	Y	Y	8/8	Low	Large sample, standardized diagnosis, questionnaire domains, and multivariable prediction model.
Gabdulkayum, 2025 [45]	Cross-sectional genetic association; elite athletes	Y	Y	Y	Y	Y	Y	Y	Y	8/8	Low	Clear athlete sample, objective 25(OH)D assay, genotyping, and analytical strategy.
Madiyeva, 2025 [35]	Cross-sectional; healthy children	Y	Y	Y	Y	Y	Y	Y	Y	8/8	Low	Large sample, laboratory assessment, questionnaire domains, and multivariable prediction model.
Madiyeva, 2025 (b) [46]	Cross-sectional; adults with somatic diseases	Y	Y	Y	Y	Y	Y	Y	Y	8/8	Low	Clear adult sample, laboratory assessment, questionnaire domains, and multivariable prediction model.

Abbreviations: Y, yes; N, no; U, unclear; JBI, Joanna Briggs Institute.

**Table 3:** GRADE certainty-of-evidence assessment for pooled serum 25-hydroxyvitamin D levels in Kazakhstan.

Outcome	Participants	Pooled Estimate	Risk of Bias	Inconsistency	Indirectness	Imprecision	Publication Bias	Downgrading	Certainty of Evidence
Mean serum 25(OH)D level: overall cohort	5771	22.3 ng/mL (95% CI 19.3 to 25.3)	Not serious	Very serious	Not serious	Not serious	Not serious	2	Very low
Mean serum 25(OH)D level: healthy cohort	3152	24.4 ng/mL (95% CI 20.3 to 28.4)	Not serious	Very serious	Not serious	Not serious	Not serious	2	Very low

## Data Availability

The original contributions presented in this study are included in the article. Further inquiries can be directed to the corresponding authors Galiya Bilibayeva bilibayeva@kaznu.kz and Dinara Ospanova ospanova.dinara@kaznu.kz.

## Supplementary Materials

The following supporting information can be downloaded at: https://www.mdpi.com/article/10.3390/diagnostics16121851/s1, Table S1: PRISMA checklist.
